# Prevalence and genetic diversity of polymorphisms in *pfcrt*, *pfdhfr-ts* and *pfk13 propeller* genes of *Plasmodium falciparum* in southern Côte d'Ivoire

**DOI:** 10.5281/zenodo.14604138

**Published:** 2025-01-06

**Authors:** Oléfongo Dagnogo, Ako A.B. Ako, Dougba N. Dago, Kouamé B.A. Kouman, N'golo D. Coulibaly, Kouakou B. Bla, Offianan A. Touré, Allico J. Djaman

**Affiliations:** 1 Biosciences Training and Research Unit (UFR), Felix Houphouët-Boigny University, Abidjan, Côte d’Ivoire.; 2 Department of Parasitology-Mycology, Pasteur Institute of Côte d’Ivoire, Abidjan, Côte d’Ivoire.; 3 Training and Research Unit (UFR) of Biological Sciences, Peleforo Gon Coulibaly University, Korhogo, Côte d’Ivoire.

## Abstract

**Background:**

*Plasmodium falciparum* has developed resistance to almost all the antimalarial drugs currently in use. This resistance has been and remains one of the greatest threats to the control and elimination of malaria. The use of molecular markers of resistance to monitor the emergence and spread of antimalarial drug-resistant parasite strains has proved highly effective. The aim of this study was to analyse the polymorphism of the *pfcrt*, *pfdhfr-ts* and *pfK13 propeller* genes for resistance in *P. falciparum* to chloroquine (CQ), pyrimethamine and artemisinin-based combination therapies (ACTs) in three sites in southern Côte d'Ivoire.

**Methodology:**

Blood samples were collected in Anonkoua-kouté, Port-Bouët, and Ayamé from 94 patients with microscopically confirmed uncomplicated *P. falciparum* malaria. These patients, aged over 2 years, gave their informed consent prior to blood sampling. *P. falciparum* genomic DNA extracted from these samples was amplified by nested PCR using primers specific to the *pfcrt*, *pfdhfr-ts* and *Pfk13 propeller* genes. The amplification products were sequenced using the Sanger method. After sequencing, the prevalence of *pfcrt* (M74I, N75E, K76T), *pfdhfr* (N51I, C59R, S108N) and *pfk13 propeller* (Y493H, R539T, I543T, C580Y, M476I and R561H) mutations confirmed to be involved in *P. falciparum* resistance to CQ, pyrimethamine and ACTs, respectively was determined. Data were analysed using R statistical software, version 3.2.2.

**Results:**

For all three study sites, 93 (93/94, i.e. 98.94%), 86 (86/94, i.e. 94.49%) and 74 (74/94, i.e. 78.72%) DNA fragments from patient isolates were successfully amplified for the *Pfk13 propeller*, *pfdhfr-ts* and *pfcrt* genes, respectively. Of the successfully amplified fragments, 93 (93/93, i.e. 100%), 81 (81/86, i.e. 94.18%) and 64 (64/74, i.e. 86.48%) were successfully sequenced for the *pfk13 propeller*, *pfdhfr-ts* and *pfcrt* genes, respectively. Sequence analysis indicated that S108N mutations in the *pfdhfr* gene and K76T mutations in the *pfcrt* gene were observed in 74.07% (60/81) and 15.62% (10/64) respectively. Analysis of the *k13 propeller* gene also showed a predominance of the YRICMR allelic form representing the sensitive haplotype (72/93, i.e. 78.49%).

**Conclusions:**

More than a decade after the abandonment of the use of CQ and the adoption of sulfadoxinepyrimethamine (SP) as intermittent preventive treatment (IPT) for pregnant women, the prevalence of alleles associated with CQ chemoresistance, represented by the K76T mutation in the *pfcrt* gene, fell, while that of alleles associated with pyrimethamine chemoresistance, represented by the S108N mutation in the *pfdhfr-ts* gene, increased in Anonkoua-Kouté, Port-Bouët and Ayamé. No mutations in mutant alleles of the *K13 propeller* gene conferring resistance to artemisinin derivatives were observed at any of the study sites. The study thus showed that the ACTs used for first-line treatment of malaria in Côte d'Ivoire are still effective.

## Introduction

Malaria is one of the deadliest infectious diseases in tropical and subtropical regions in terms of morbidity and mortality. The World Health Organization (WHO) has formulated the Malaria Control Strategy with the vision of a malaria-free world by 2030 [1].

According to the WHO, around 249 million cases of malaria were recorded in 2022, of which 610,000 caused deaths, of which around 580,000 (95%) occurred in Africa [2]. The vast majority of deaths (78%) occurred in children under five, with 452,000 deaths in 2022, or 74% of all malaria deaths [2]. Despite efforts to combat malaria, the disease remains a serious public health problem. The emergence of *P. falciparum* resistance to almost all available antimalarial drugs is making the situation more complicated and difficult. South-East Asia (SEA) is considered the epicentre for the evolution and spread of resistance against all major classes of antimalarials [3]. Resistance to CQ and SP in *P. falciparum* emerged in the late 1950s and 1960s on the Thai-Cambodian border and spread across Asia and then Africa, resulting in millions of malaria deaths [4,5]. In response to this situation, the WHO has recommended the use of ACTs as first-line treatment for uncomplicated *P. falciparum* malaria in all malaria-endemic countries [6]. Since 2005, Côte d'Ivoire has adopted new disease control strategies, which involve the use of ACTs for all confirmed cases of malaria in both adults and children. This strategy included the free dispensing of ACTs for confirmed cases of malaria in children under 5 and pregnant women.

However, the official introduction of ACTs has led to fears of widespread use of cheap CQ and SP, which could increase *P. falciparum* resistance to CQ and SP and reduce sensitivity to ACTs. To ensure early warning and better management of malaria in Côte d’Ivoire, it is necessary to set up a system for monitoring *P. falciparum* resistance to CQ, pyrimethamine and artemisinin derivatives. The efficacy of antimalarial drugs can be determined by four different methods: *in vivo* therapeutic efficacy studies, *in vitro* tests, molecular marker studies and the measurement of drug concentrations. The *in vivo* therapeutic efficacy study is still considered the gold standard in this respect. Molecular marker studies are also important for determining any early signs of antimalarial drug resistance. Several studies have conclusively shown a correlation between the development of antimalarial drug resistance and the presence of polymorphisms in the genes of the *P. falciparum* parasite that determine the effects of the drugs [7,8]. For example, mutations in the *pfcrt* gene encompassing codons 72-76 have been associated with CQ resistance in *P. falciparum*, while the K76T mutation is characteristic of CQ resistance [7,9]. Furthermore, the N86Y mutation in the *pfmdr* gene of *P. falciparum* modulates a higher level of resistance to CQ when present with the K76T mutant in the *pfcrt* gene [10]. In addition, point mutations in the *pfdhfr* and *pfdhps* genes confer resistance to pyrimethamine and sulfadoxine respectively [10-12] with an *in vitro* decrease in *P. falciparum* susceptibility linked to the number of mutations in each gene. Polymorphisms at codons 436, 437, 540, 581 and 613 of *pfdhps* and at codons 16, 51, 59, 108 and 164 of the *pfdhfr* gene have been identified as markers of resistance to sulfadoxine and pyrimethamine [7]. Artemisinin resistance is characterized by slow elimination of the parasite [13], which reflects the reduced susceptibility of the asexual stages of the parasites. [10,14]. This resistance of *P. falciparum* to artemisinin derivatives reported in various countries [14,15] has been correlated with point mutations in the *pfK13 propeller* gene [14]. Periodic monitoring of polymorphisms in molecular markers associated with antimalarial drug resistance will be useful to assess drug pressure, map and monitor the resistance status of these drugs; and will be useful to search for alternative treatments. This study was conducted in three localities in southern Côte d'Ivoire to determine the prevalence of key mutations associated with *P. falciparum* resistance to CQ, pyrimethamine and artemisinin derivatives in subjects with uncomplicated malaria.

## Materials and Methods

### Study site

Samples were collected from February to August 2015 at the Anonkoua Kouté health center and the Port-Bouët and Ayamé general hospitals. All these sites are located in the southern region of Côte d'Ivoire, where the climate is Atrean, with annual rainfall in excess of 1,700 mm and temperatures ranging from 27 to 33°C. Malaria is seasonal, predominating in the rainy season from June to September, with peaks in prevalence and incidence in October-November. *P. falciparum* is the dominant species, accounting for over 90% of the parasite formula. The main malaria vectors in this study area (the forested south of Côte d'Ivoire) are members of the *An. gambiae s.l.* and *An. funestus s.l.* complexes [16]. The Anonkoua-kouté health center and the Ayamé general hospital were selected because of the high annual incidence of malaria cases. In addition, these health facilities have been considered for several years as the main sites for multicenter clinical efficacy testing by the Malaria Unit of the Pasteur Institute of Côte d'Ivoire. The Port Bouët General Hospital was chosen for this study not only because of its consistently high annual incidence of malaria, but also and above all because of its marshy environment used for market gardening.

### Study population and sample collection

All patients at the Anonkoua-kouté health center and the Port-Bouët and Ayamé general hospitals with clinical symptoms suggestive of malaria during our study period were eligible for blood sampling after obtaining informed consent. Following informed consent, blood samples were collected from patients aged over 2 years with an axillary or rectal temperature above 37.5°C and uncomplicated *P. falciparum* malaria confirmed microscopically by thick drop and a blood smear.

### Blood sampling

From each patient with microscopically confirmed malaria, approximately 2-5 mL of venous blood was drawn and collected in an EDTA tube. Approximately 50 μL of whole blood was spotted onto Whatman 3 mm filter paper using a micropipette with filter cones. The paper containing the blood spots was dried for approximately 60 to 120 minutes at room temperature in a dust-free environment. Unused blood from the EDTA tube was stored in cryotubes at -20°C for possible future use.

### Extraction of *Plasmodium falciparum* genomic DNA

Plasmodium DNA was extracted with methanol from blood confetti [17]. Thin cuts of blood confetti were immersed in 1 mL of wash buffer (950 μL of 1X PBS plus 50 μL of 10% saponin) and then incubated overnight at 4°C. The wash buffer was removed and washed before adding 150 μL of methanol. After incubation for 20 minutes, the methanol was gently removed and the samples were dried at room temperature for 2 hours before adding 300 μL of sterile water. The samples were then heated to 99°C in a thermo-mixer for 30 minutes to elute the DNA. After removing the confetti debris, the DNA extracts were aliquoted into a 1.5 mL Eppendorf tube and stored at -20°C.

### Amplification of the *pfcrt*, *pfdhfr* and *pfk13 propeller* genes

The *pfcrt*, *pfdhfr* and *pfk13 propeller* resistance genes were amplified by nested PCR using a pair of primers specific for each gene and a commercial DNA polymerase kit called 5X FIREPol® Blend Master Mix with mM MgCl2.

The composition of this kit constituted a pre-mix for the reaction mixture. For the primary PCR, the primer pairs used for the *pfk13 propeller*, *pfdhfr* and *pfcrt* genes were respectively K13_PCR_F(5'CGGAGTGACCAAATCTGGGA)/ K13_PCR_R(5'GGGAATCTGGTGGT AACAGC), dhfr_M1(5'TTTATGATGGAACAAGTCTGC) / dhfr_M7(CTAGTATATACATCGC TAACA) and 72_97EF(5' GAC CTT AAC AGA TGG CTC AC) / 72_97ER(5' TTT TAT ATT GGT AGG TGG AAT AG). Primary PCR of these genes was performed in a 25 μl reaction volume containing: 0.625 μL of each primer, 3 μL of plasmodial DNA, 5 μL of *Taq* polymerase and 15.75 μL of milliQ water. The mixture was then placed in a PTC-100TM thermal cycler (Eppendorf Mastercycler, PTC-100 Peltier Thermal Cycler), programmed as follows: Initial denaturation at 95°C for 15 minutes followed by 30 cycles of denaturation at 95°C for 30 seconds, hybridisation at 58°C for 2 minutes and extension at 72°C for 2 minutes. Finally, a terminal extension at 72°C for 10 minutes.

The second PCR was performed on the amplification products of the primary PCR in a 50 μl reaction volume containing: 1.25 μL of each primer, 5 μL of amplification product (amplifiate) from the first PCR, 5 μL of *Taq* polymerase and 37.5 μl of milliQ water.

The primer pairs used for the secondary PCR were K13_N1_F(5'GCCAAGCTGCCATTCATTTG)/ K13_N1_R(5'GCCTTGTTGAAAGAAGCAG) for the *pfk13 propeller* gene, dhfr_M9(5' CTG-GAAAAAATACATCACATTCATATG) / dhfr_M3 (5' TGATGGAACAAGTCTGCGACGTT) for the *pfdhfr* gene and SecIF (5' GGTAAATGTGCTCAT-G T G T T T A A A C T T A T T ) / S e c I R (5’TTACTTTTGAATTTCCCTTTAT TTCCA).

Secondary PCR was performed using the same thermal cycler used for primary PCR with the following programme: Initial denaturation at 95°C for 15 minutes followed by 30 cycles of denaturation at 95°C for 30 seconds, hybridisation at 60°C for one minute and extension at 72°C for one minute. Finally, a terminal extension at 72°C for 10 minutes.

### Detection and analysis of PCR products

The amplification products of the *pfcrt*, *pfdhfr* and *pfK13 propeller* genes were migrated onto a 1.5% (W/V) agarose gel containing Ethidium Bromide (BET). After migration, the gel was recovered and observed under a UV lamp using the UV transluminator (Gel DocTM EZ Imager). The presence or absence of bands was used to judge PCR efficiency.

### Sequencing amplification

In this work, the amplified DNA fragments (*pfcrt*, *pfdhfr* and *pfK13 propeller* genes from *P. falciparum*) were sequenced using the Sanger method by Eurofins MWG opéron (Cochin sequencing platform). Samples were supplied to the platform in a microplate (Greiner Bio-one-652270B) accompanied by a deposit slip which was sent to the platform's email address. A reaction medium was prepared for the nested PCR sense primer (sequencing primer) from the amplification products.

In each well of the microplate, a volume of 13 μL of amplification product was added to 2 μL of 10 μM sequencing primer. The wells containing the sequencing reaction medium were sealed with cap strips (4titude-044737) before covering the entire surface of the microplate with adhesive film (AmpliSeal, Greiner Bio-one-676040). This microplate containing our samples was supplied to the platform for sequencing. After the sequencing reaction, the DNA sequences received were recovered in fasta form. In the case of this work, these were the sequences corresponding to the *pfcrt*, *pfdhfr* and *pfK13 propeller* genes of the isolates collected. The sequences were analysed for mutations using BioEdit software. Codons of interest (74, 75, 76) in the PFCRT polypeptide or nucleotides in positions 222, 225, 228 of the *pfcrt* gene sequence were identified and analysed after parallel alignment of two or more DNA sequences, including the reference sequence of the *pfcrt* gene, maximising the number of identical nucleotides or residues, while minimising the number of mismatches and gaps. Codons 51, 59 and 108 of the PfDHFR polypeptide and nucleotides at positions 153, 177 and 324 of the *pfdhfr* gene were also analysed. Codons 493, 539, 543, 580, 476 and 561 of the PFK13 PROPELLER polypeptide and nucleotides at positions 1479, 1617, 1629, 1740, 1428 and 1683 of the *K13 propeller* gene were also analysed.

### Statistical analysis of data

Data were collected using a standard questionnaire that had been tested and validated. They were then entered and analysed on R statistical software; version 3.2.2 [18]. The χ2 test of comparison of three means was used to compare the prevalences of molecular markers of resistance to CQ (*pfcrt* K76T), pyrimethamine (*pfdhfr* S108N). The χ2 test was used to determine whether the molecular marker prevalences can be considered to be all equal (null hypothesis H0) or whether at least two prevalences are different (alternative hypothesis Ha). A statistical difference and/or association was considered significant if p of the χ2 test < 0.05.

## Results

### Patient profile and selected isolates

A total of 94 people infected with *P. falciparum* were included in the study, 58 (61.7%) of them women and 36 (38.3%) men. The patients ranged in age from 2 to 62 years, with an average age in Anonkoua-kouté, Port-bouët and Ayamé of 16.60, 16.69 and 15.84 years, respectively. A total of 94 blood samples were collected from the three study sites (Table 1).

**Table 1:** Samples used for molecular analysis of chemoresistance to chloroquine, pyrimethamine and artemisinin derivatives.

Sites	Period of collections in 2015	Brackets age (years)	Average age (years)	Conffetis collected
Anonkoua- kouté	February - March	2 - 53	16,60	30
Port - Bouët	April - May - July	2 - 62	16,69	32
Ayamé	June - July - August	2 - 55	15,84	32
**Total**				**94**

### Amplification and sequencing results

Across the three study sites, 94 DNA fragments from patient isolates were amplified, including 93 (93/94, 98.84%) fragments of the *pfk13 propeller* gene, 86 (86/94, 94.49%) of the *pfdhfr* gene and 74 (74/94, 78.72%) of the *pfcrt* gene successfully amplified (Table 2).

**Table 2:** PCR sensitivity for the genes studied.

	Amplified DNA fragments (N* = 94)		
	*K13 propeller*	*pfdhfr*	*pfcrt*
Amplification success n** (%)	93 (98,84)	86 (94,49)	74 (78,72)

* N represents the number of isolates amplified.

**n represents the number of isolates successfully amplified per gene.

Molecular analysis of the amplified fragments showed that the number of DNA fragments successfully sequenced varied according to the presence of codons of interest on the DNA fragment of the amplified gene. Of the 86 DNA fragments of the *pfdhfr* gene sequenced, 65 (75.60%), 66 (76.75%) and 81 (94.20%) were successfully sequenced for nucleotides at positions 153, 177 and 324, respectively, corresponding to the amino acids at which the Asn-51-Ile, Cys-59-Arg and Ser-108-Asn mutations were observed (Table 3). Sequencing of the DNA region leading to the Ser-108-Asn mutation was more successful (155/165; 93.93%).

**Table 3:** Summary of sequencing of DNA extracted from isolates according to mutations.

Sequenced frag- ments (N*= 94)	Changes	Success n**(%)	Ches n** (%)
		Blood (n=86)	
	Asn-51-Ile	65 (75,60%)	21 (24,4%)
*Pfdhfr* (N*=86)	Cys-59-Arg	66 (76,75%)	20 (23,25%)
	Ser-108-Asn	81 (94,20%)	**5** (5,80%)
		Blood (n=74)	
	Met-74-Ile	59 (79,73%)	15 (20,27%)
*Pfcrt* (N*=74)	Asn-75-Glu	64 (84,50%)	10 (13,50%)
	Lys-76-Thr	64 (84,50%)	**10** (13,50%)
		Blood (n=93)	
	Tyr-493-Ile	79 (84,94%)	14 (15,06%)
*pfk13 propeller* (N*=93)	Arg-539-Thr	85 (91,40%)	8 (8,60%)
	Ile-543-Thr	89 (95,70%)	4 (4,30%)
	Cys-580-Tyr	93 (100%)	**0** (0,0%)
	Met-476-Ile	77 (82,80%)	16 (17,20%)
	Arg-561-His	88 (94,60%)	5 (5,40%)

*N represents the total number of isolates successfully sequenced at the three sites.

**n represents the number of isolates for which codons of interest of the sequence were located.

Similarly, out of 74 DNA fragments of the *pfcrt* gene, 59 (79.73%), 64 (84.50%) and 64 (84.50%) DNA fragments were successfully sequenced for nucleotides at positions 222, 225 and 228 respectively, corresponding to the amino acids at which the Met-74-Ile, Asn-75-Glu and Lys-76-Thr mutations were observed.

For the *pfk13 propeller* gene, of the 93 DNA fragments successfully sequenced, 79 (84.94%), 85 (91.40%), 89 (95.70%), 93 (100%), 77 (82.80%) and 88 (94.60%) were successfully sequenced for nucleotides at positions 1479, 1617, 1629, 1740, 1428 and 1683 respectively, corresponding to the amino acids at which the Tyr-493-Ile, Arg-539-Thr, Ile-543-Thr, Cys- 580-Tyr, Met-476-Ile and Arg-561-His mutations were observed (Table 3).

Sequencing of the DNA region leading to the Cys-580-Tyr mutation was more successful (186/186; 100%).

### Polymorphism of *pfdhfr* and *pfcrt* genes in the study sites

#### Prevalence of individual alleles of pfdhfr and pfcrt genes

For all three study sites, our results indicate that the prevalences of isolates carrying the Ile-51 (61.29%), Arg-59 (54.76%) and Asn-108 (74.19%) mutations are higher than those of wild-type Asn-51 (15.32%), Cys-59 (15.07%) and Ser-108 (17.41%) isolates of the *pfdhfr* gene (Table 4).

**Table 4:** Prevalences of individual alleles of *pfdhfr* and *pfcrt* genes in the study sites.

Genes	Codons	Alleles		n*	Frequencies
				n=65	(%)
*pfdhfr*	dhfr_51	Wild (N)	Asn-51	10	15,38
			Ile-51	40	61,53
		Mutants	Other	15	23,07
				n=66	(%)
	dhfr_59	Wild (C)	Cys-59	10	15,15
			Arg-59	36	54,54
		Mutants	Other	20	30,30
				n=81	(%)
	dhfr_108	Wild (S)	Ser-108	14	17,28
			Asn-108	60	74,07
		Mutants	Other	7	8,64
				n=59	(%)
*pfcrt*	pfcrt_74	Wild (M)	Met-74	47	73,43
		Mutants	Ile-74	5	7,81
			Other	1	1,56
				n=64	(%)
	pfcrt_75	Wild (N)	Asn-75	48	75
			Glu-75	5	7,81
		Mutants	Other	6	9,37
				n=64	(%)
	pfcrt_76	Wild (K)	Lys-76	40	62,50
			Thr-76	10	15,62
		Mutants	Other	1	1,56

n* represents the number of isolates for which *pfdhfr-*ts and *pfcrt* genes codons of interest (51, 59, 108 and 74, 75, 76 respectively) or nucleotides at respective positions 153, 177, 324 and 222, 225, 228 of sequence were identified. The list of other mutants can be found in Supplementary Tables (Appendix 1 for *pfcrt* and Appendix 3 for *pfdhfr-ts*).

Similarly, for the *pfcrt* gene, the prevalences of wild isolates Met-74 (73.43%), Asn-75 (75%) and Lys-76 (62.5%) are higher than those of isolates carrying the Ile-74 (7.81%), Glu-75 (7.81%) and Thr-76 (15.62%) mutations (Table 4).

### Molecular analysis of genotypes corresponding to pfdhfr and pfcrt genes

Molecular analysis of the genotypes corresponding to the *pfdhfr* gene showed that isolates carrying the IRN (triple mutant haplotype), NRN (double mutant haplotype) and ICN (double mutant haplotype) genotypes were observed with respective prevalences of 31.40% (27/86), 9.30% (8/86) and 8.14% (7/86) compared with 13.93% for the NCS susceptible haplotype (Table 5). The analysis also revealed a predominance of the sensitive MNK haplotype of the *pfcrt* gene with a prevalence of 62.5% (40/64) compared with 6.25% (4/64) and 9.37% (6/64) for the IET and LKQ mutant haplotypes respectively. Single mutant (SNM) and double mutant (NYQ) haplotypes were observed at prevalences of 9.37% (6/64) and 6.25% (4/64), respectively (Table 5).

**Table 5:** Prevalences of haplotypes of *pfcrt*, *pfdhfr* genes.

		Allelic forms				
Gene	Haplotypes	M74I	N75E	K76T	n	Prevalences
*pfcrt* (N* = 64)	Wild	M	N	K	40	62,5
	Simple mutants				8	12,5
		M	N	T	6	9,37
		Other			2	3,12
	Double mu-	N	Y	Q	4	6,25
	Triple mutants				12	18,75
		I	E	T	4	6,25
		L	K	Q	6	9,37
		Other			2	3,12
**Gene**	**Haplotypes**	**N51I**	**C59R**	**S108N**	**n**	**Prevalences**
*pfdhfr* (N* = 86)	Wild	N	C	S	12	13,96
	Simple mutants				9	10,47
		N	C	N	2	2,33
		Other			7	8,13
	Double mu-				26	30,23
		N	R	N	8	9,30
		I	C	N	7	8,13
		Other			11	12,79
	Triple mutants				39	45,34
		I	R	N	27	31,40
		Other			12	13,95

N* represents the total number of isolates successfully sequenced at the three sites. A capital letter in the allelic forms column represents the single-letter code for the amino acids. The amino acids resulting from the mutation of *pfcrt* and *pfdhfr* are underlined and in bold. The frequencies determined correspond to the number of observations out of the number of hits per gene. The list of other mutants can be found in Supplementary Tables (Appendix 2 for *pfcrt* and Appendix 4 for *pfdhfr-ts*).

### Polymorphism of the *pfk13 propeller* gene in the study sites

#### Prevalence of individual alleles of the pfk13 propeller gene

For all three study sites, our results indicate that the prevalences of isolates carrying the Ile-493 (1.26%), Thr-539 (3.52%) and Ile-476 (1,29%) are very low compared to the wild-type isolates Tyr-493 (92.40%), Arg-539 (94.11%), Ile-543 (94.38%), Cys-580 (91.39%), Met-476 (89.61%) and Arg-561 (88.63%) of the *pfk13 propeller* gene (Table 6).

**Table 6:** Prevalence of individual alleles of the *pfk13 propeller* gene.

			Study sites (N*=93)
Codons	Strains and mutations observed		Number of employees (%)
K13_493 (n** = 79)	Wild	Tyr-493	73 (92,40)
	Mutants	His-493	1 (1,26)
		Pro-493	1 (1,26)
		Phe-493	3 (3,79)
		Cys-493	1 (1,26)
K13_539 (n** = 85)	Wild	Arg-539	80 (94,11)
	Mutants	Thr-539	3 (3,52)
		Gly-539	1 (1,17)
		Pro-539	1 (1,17)
K13_543 (n** = 89)	Wild	Ile-543	84 (94,38)
	Mutants	Thr-543	0,00
		Met-543	2 (2,24)
		Phe-543	2 (2,24)
		Ser-543	1 (1,12)
K13_580 (n** = 93)	Wild	Cys-580	85 (91,39)
	Mutants	Tyr-580	0,00
		Ser-580	3 (3,22)
		Pro-580	3 (3,22)
		Gly-580	1 (1,07)
		Arg-580	1 (1,07)
K13_476 (n** = 77)	Wild	Met-476	69 (89,61)
	Mutants	Ile-476	1 (1,29)
		Gly-476	2 (2,59)
		Lys-476	1 (1,07)
		Arg-476	2 (2,59)
		Thr-476	1 (1,29)
		Ser-476	1 (1,29)
R561H	Wild	Arg-561	78 (88,63)
	Mutants	His-561	0,00
		Cys-561	7 (7,95)
		Trp-561	1 (1,13)
		Ser-561	1 (1,13)
		Leu-561	1 (1,13)

*N represents the total number of isolates successfully sequenced at the three sites.

**n represents the number of isolates for which codons of interest (493, 539, 543, 580, 478 and 561) or nucleotides at positions 1479, 1617, 1629, 1740, 1428 and 1683 of the sequence were identified.

The Thr-543, Tyr-580 and His-561 mutations were not observed. However, isolates carrying the Cys-561 mutation (7.95%) were observed. Very low prevalences (less than 5%) of other mutations were also detected.

#### Molecular analysis of genotypes corresponding to the pfk13 propeller gene

Molecular analysis of the genotypes corresponding to the *pfk13 propeller* showed that the YRICMR allelic form (susceptible haplotype) predominated in the isolates with a prevalence of 77.42% compared with 7.53%, 9.68%, 3.22% and 2.15%, respectively for the single mutant, double mutant, triple mutant and quadruple mutant haplotypes observed (Table 7).

**Table 7:** Prevalences of genotypes corresponding to *pfk13 propeller* in the three sites.

								Blood (N= 93)
Haplotype	Y493H	R539T	I543T	C580Y	M476I	R561H	n	Proportion
**Wild**	Y	R	I	C	M	R	72	77,42
Simple Mutants							7	7,53
	Y	R	I	C	K	R	1	
	Y	R	I	C	R	R	1	
	Y	R	I	C	T	R	1	
	Y	R	I	C	M	C	1	
	C	R	I	C	M	R	1	
	Y	R	I	G	M	R	1	
	Y	R	I	S	M	R	1	
**Double Mutants**							9	9,68
	Y	R	I	C	G	C	1	
	Y	G	I	S	M	R	1	
	P	R	I	C	M	C	1	
	Y	R	M	C	M	C	1	
	Y	R	F	P	M	R	1	
	F	T	I	C	M	R	1	
	F	R	I	C	R	R	1	
	Y	R	I	C	S	C	1	
	Y	R	I	R	M	L	1	
**Triple Mutants**							3	3,22
	H	R	I	C	I	C	1	
	Y	R	M	P	M	W	1	
	Y	P	F	F	M	R	1	
**Quadruple Mutants**							2	2,15
	Y	T	S	P	M	S	1	
	F	T	I	C	G	C	1	

A capital letter in the genotypes column represents the single-letter code for the amino acids. The amino acids resulting from the mutation are underlined and in bold. The prevalences determined correspond to the number of observations out of the number of hits per gene.

## Discussion

The use of molecular biomarkers is essential for identifying and understanding the genetic mutations responsible for *P. falciparum* resistance to antimalarial drugs. This study was carried out to analyse the polymorphism of the *pfcrt*, *pfdhfr-ts* and *pfK13 propeller* genes conferring resistance in *P. falciparum* to CQ, pirymethamine and ACTs respectively in three sites in southern Côte d'Ivoire. DNA fragments from isolates from patients at the three study sites were amplified and sequenced. Polymorphism analysis of the genes studied (*pfk13 propeller*, *pfdhfr-ts* and *pfcrt*) was carried out. With regard to the *pfdhfr-ts* gene, the results indicate that the Ser-108-Asn (Asn-108) mutation was observed in 74.07% of the three study sites. The high prevalence of this mutation could be explained by the presence of potentially pyrimethamine-resistant *P. falciparum* isolates.

This prevalence is higher than those obtained by Ako *et al.* [19] in 2014 at Anonkoua-Kouté in Abidjan (49%) and Ayamé (54%) in blood isolates from individuals with malaria symptoms. Lower proportions had already been observed in 2010 in the south of Côte d'Ivoire by Djaman *et al.* [20] in Yopougon (Abidjan) and Ouattara *et al.* [21] in Adzopé. Our results corroborate those of Ako *et al.* [19,22] who indicated that the prevalence of the Ser-108-Asn mutation had increased significantly in Anonkoua-kouté between 2002 and 2008 with an average of 43%. All these results clearly indicate that the prevalence of the Asn-108 mutation has increased significantly in this part of the country. This finding is also important because the SP combination is still in circulation because it is recommended for intermittent preventive treatment in pregnant women in Côte d'Ivoire [23]. The results obtained could therefore be explained by increased use of SP on parallel markets as a result of the effective withdrawal of CQ. This drug pressure could be at the origin of the circulation of pyrimethamine-resistant strains in the three study sites, with an increase in the prevalence of Ile-51 (61.29%), arg-59 (54.76%) and Asn-108 (74.19%) mutations in the *pfdhfr-ts* gene in the three study sites. Indeed, these mutations are fixed in the parasite and confer resistance to pyrimethamine, implying that the efficacy of the SP combination as Intermittent Preventive Treatment would be limited in this region [24]. In addition to drug pressure, pyrimethamine resistance in these three localities could be explained by the use of poor-quality antimalarials. Indeed, the use of poor quality antimalarials can have multiple consequences, including an increased risk of developing drug resistance, as sub-therapeutic doses of drugs will be ineffective in destroying all parasites [25,26].

Elsewhere in sub-Saharan Africa, high rates have been found. Monitoring of *P. falciparum* chemoresistance has yielded the following results: in Burkina Faso, the reported rate of the Asn-108 mutation was 63.8% [27], 66.9% in Cameroon [7] and 64% in Benin [28]. As well as the Asn-108 mutation, additional mutations (Asn-51-Ile and Cys-59-Arg) were identified. All the mutations at codons 51 and 59 were associated with codon 108. Parasites carrying these additional Asn-51-Ile and Cys-59-Arg mutations associated with the Ser-108-Asn mutation have 2000 times greater resistance to pyrimethamine than those carrying the Ser-108-Asn mutation alone [29,30].

With regard to the *pfcrt* gene, previous studies have shown a marked correlation between the Thr-76 mutation in the *pfcrt* gene and therapeutic failures on the one hand, and between the Thr-76 mutation in the *pfcrt* gene and *in vitro* chemoresistance of *P. falciparum* isolates to CQ on the other [31-33].

Our results indicate that across all three study sites, the Thr-76 mutant allele (15.63%) was associated with the Ile-74 (7.81%) and Glu-75 (7.81%) mutant alleles in isolates at very low proportions compared with the wild-type alleles Lys-76 (62.50%), Met-74 (73.43%) and Asn-75 (75%).

In addition, the wild Lys-76 allele was observed in Anonkoua-Kouté, Port-Bouët and Ayamé at 60.71%, 62.50% and 65% respectively. These results are similar to those of Kouman *et al.* [34], who observed a predominance of the wild 76-Lys allele at five sites in Côte d'Ivoire, with a prevalence ranging from 60.9 to 86.4%.These high prevalences of the wild Lys-76 allele could be explained by the drop in drug pressure associated with the effective withdrawal of CQ in Côte d'Ivoire. When drug pressure is low, drug resistance is accompanied by a reduction in the genetic performance of resistant parasites compared with susceptible parasites [35,36]. Thus, when drug pressure is reduced, the proportion of susceptible parasites increases and that of resistant parasites decreases [37]. Thus, the withdrawal of CQ and the introduction of ACTs seem to favour a re-emergence of CQ-susceptible isolates in Côte d'Ivoire. Elsewhere in malaria-endemic areas, similar frequencies were observed for the wild allele of the *pfcrt* gene after CQ was abandoned for the treatment of malaria. In Burkina Faso, Nigeria and Equatorial Guinea, the reported prevalence of the wild Lys-76 allele was 63.8%, 63.9% and 74% respectively [27,38,24].

Thus, the withdrawal of CQ and the introduction of ACTs seem to favour a re-emergence of CQ-susceptible isolates. It would be desirable to carry out another study covering several localities with a larger number of samples to confirm this decrease in CQ-resistant parasites in Côte d'Ivoire. Thus, if the proportion of CQ-resistant parasites decreases at national level to an undetectable level of *pfcrt* mutants, the reintroduction of CQ in combination with other antimalarial drugs for treatment and prophylaxis could be envisaged, as has been done in Malawi [37].

Polymorphism analysis of the *k13 propeller* gene indicates very low proportions of mutations associated with *P. falciparum* resistance to artemisinin derivatives. Indeed, for the three study sites, the analysis showed that the YRICMR allelic form (sensitive haplotype) was predominant, with a prevalence of 78.49%. In addition, this analysis indicated prevalences of 1.26%, 3.52% and 1.29% respectively for the His-493, Thr-539 and Ile-476 mutant alleles, compared with 92.40% (His-493), 94.11% (Thr-539), 94.38% (Thr-543), 91.39% (Tyr-580), 89.61% (Ile-476) and 88.63% (His-561) for the wild-type alleles. No mutations in the Ile-543, Cys-580 or His-561 alleles were observed at any of the three sites studied. Overall, our results indicate a very low prevalence of mutations associated with resistance to artemisinin derivatives. These low proportions of known mutations in the *pfK13 propeller* gene support the efficacy of ACTs in Côte d'Ivoire.

In Côte d'Ivoire, ACTs have been recommended as first-and second-line treatments for uncomplicated malaria since 2005, and the efficacy of these combinations remains high, as reported by Touré *et al.* [39,40]. The use of ACTs increased in the country after the adoption of their free distribution to children under the age of five. Consequently, drug pressure due to uncontrolled use (prescription or self-medication) of ACTs could have selected resistant parasites over time. It is also important to monitor the possible emergence of a population of ACT-resistant parasites. However, these results need to be confirmed by sequencing the complementary DNA strand and also by *in vitro* tests such as the RSA (Ring-stage Survival Assay).

## Conclusion

The main objective of this study was to analyse the polymorphism of the *P. falciparum pfcrt*, *pfdhfr* and *pfK13 propeller* genes in patients with uncomplicated malaria. To do this, the prevalence of mutations in the *pfcrt*, *pfdhfr* and *pfK13 propeller* genes involved in resistance to CQ, SP and ACT respectively, was determined. The study showed that more than a decade after the discontinuation of CQ use in Côte d’Ivoire and the adoption of SP as intermittent preventive treatment (IPT) for pregnant women, the prevalence of alleles associated with CQ chemoresistance, represented by the K76T mutation in the *pfcrt* gene, fell, while that of alleles associated with pyrimethamine chemoresistance, represented by the S108N mutation in the *pfdhfr-ts* gene, increased in Anonkoua-Kouté, Port-Bouët and Ayamé. No mutations in mutant alleles of the *K13 propeller* gene conferring resistance to artemisinin derivatives were observed at any of the study sites. The study thus showed that the ACTs used for first-line treatment of malaria in Côte d'Ivoire are still effective.

## Abbreviations

*pfcrt*: *Plasmodium falciparum* Chloroquine Resistance Transporter
*pfdhfr-ts*: *Plasmodium falciparum* Dihydrofolate Reductase Thymidylate Synthetase
*pfk13 propeller*: *Plasmodium falciparum* Kelch13 Propeller
PCR: Polymerase Chain Reaction
DNA: Deoxyribonucleic Acid
EDTA: Ethylene Diamine Tetra-Acetic Acid

## Appendix

**Appendix 1:** Frequency of other mutant alleles of the *pfcrt* gene in blood

			Blood (N=74)	
Codons	Strains and mutations observed		Numbers	Proportions (%)
			Blood (n=59)	
Crt_74	Wild type	Met-74	47	79.66
	Mutants	Ile-74	5	8.47
		Lys-74	1	1.69
		Leu-74	5	8.47
		Pro-74	0	0
		Trp-74	1 (1,69)	1.69
			Blood (n=64)	
Crt_75	Wild type	Asn-75	48	75.00
	Mutants	Glu-75	5	7.81
		Lys-75	6	9.38
		Gln-75	0	0
		Tyr-75	5	7.81
			Blood (n=64)	
Crt_76	Wild type (K)	Lys-76	40	62.50
	Mutants	Thr-76	10	15.63
		Gly-76	1	1.56
		Ile-76	1	1.56
		Glu-76	12	18.75

**Appendix 2:** Frequency of other mutant genotypes corresponding to the *pfcrt* gene

	Genotypes			Blood (N=64)	
Haplotypes dhfr	M74I	N75E	K76T	n	Proportions
Wild type (WT)	N	N	K	40	62,5
Single mutant haplotypes (SM)				8	12,5
	N	N	T	6	9,37
	M	N	Q	2	3,16
	M	N	G	0	0
Double mutant haplotypes (DM)	N	Y	Q	4	6,25
Triple mutant haplotypes (TM)				12	18,75
	L	K	Q	6	9,37
	I	E	T	4	6,25
	K	E	G	1	1,56
	I	Y	I	1	1,56
	P	Q	E	0	0

**Appendix 3:** Frequency of other mutant alleles of the *pfdhfr-ts* gene in blood

			Blood (N=86)	
Codons	Strains and mutations observed		Numbers	Proportions (%)
			Blood (n=65)	
dhfr_51	Wild type (N)	Asn-51	10	15,38
	Mutants	Ile-51	40	61,50
		Phe-51	7	10,76
		Lys-51	1	1,53
		Leu-51	3	4,61
		Pro-51	3	4,61
		Ser-51	1	1,53
		Met-51	0	0
		Thr-51	0	0
		Val-51	0 (0)	0
			Blood (n=66)	
dhfr_59	Wild type (C)	Cys-59	10	15,15
	Mutants	Arg-59	36	54,54
		Ala-59	1	1,51
		Gly-59	11	16,66
		Leu-59	1	1,51
		Ser-59	5	7,57
		Trp-59	2	3,03
		Pro-59	0	0
		Asn-59	0	0
		Val-59	0	0
		Phe-59	0	0
		Tyr-59	0	0
			Blood (n=81)	
dhfr_108	Wild type (S)	Ser-108	14	17,28
	Mutants	Asn-108	60	74,07
		Ala-108	2	2,46
		Phe-108	1	1,23
		His-108	2	2,46
		Thr-108	1	1,23
		Val-108	1	1,23
		Asp-108	0	0
		Lys-108	0	0
		Gly-108	0	0
		Pro-108	0	0
		Ile-108	0	0
		Arg-108	0	0

**Appendix 4:** Frequency of other mutant genotypes corresponding to the *pfdhfr-ts* gene in blood

				Blood		
Haplotype	N51I	C59R	S108N	n	Proportions (%)
WT	N	C	S	12	13,96
SM				9	10,47
	N	C	T	1	1,16
	I	C	S	2	2,33
	N	C	F	1	1,16
	N	C	N	2	2,33
	F	C	S	0	0,00
	N	C	V	1	1,16
	L	C	S	1	1,16
	P	C	S	1	1,16
	N	C	D	0	0,00
	N	C	R	0	0,00
	N	C	P	0	0,00
	N	W	S	0	0,00
DM				26	30,23
	N	G	A	1	1,16
	N	S	N	2	2,33
	L	G	S	2	2,33
	F	C	N	2	2,33
	N	R	N	8	9,30
	I	C	N	7	8,14
	N	G	N	2	2,33
	N	A	A	1	1,16
	P	F	S	0	0,00
	M	R	S	0	0,00
	P	G	S	0	0,00
	F	C	G	0	0,00
	P	A	S	0	0,00
	F	C	S	0	0,00
	I	W	S	0	0,00
	I	R	S	0	0,00
	I	F	S	0	0,00
	N	P	N	0	0,00
	I	C	D	0	0,00
	T	N	S	0	0,00
	N	W	N	0	0,00
	N	Y	N	0	0,00
	P	W	S	1	1,16
TM				39	
	S	G	N	1	1
	F	L	N	1	1
	I	G	H	1	1
	F	S	N	2	2
	I	R	N	27	27
	I	R	H	1	1
	K	G	N	1	1
	I	W	N	1	1
	I	S	N	1	1
	F	G	N	2	2
	P	G	N	1	1
	P	R	R	0	0
	F	R	N	0	0
	I	L	N	0	0
	F	P	I	0	0
	F	P	N	0	0
	F	W	N	0	0
	V	R	N	0	0
	I	V	K	0	0
	V	R	K	0	0
	T	L	R	0	0
	T	F	K	0	0
